# Capturing Pain in the Cortex during General Anesthesia: Near Infrared Spectroscopy Measures in Patients Undergoing Catheter Ablation of Arrhythmias

**DOI:** 10.1371/journal.pone.0158975

**Published:** 2016-07-14

**Authors:** Barry D. Kussman, Christopher M. Aasted, Meryem A. Yücel, Sarah C. Steele, Mark E. Alexander, David A. Boas, David Borsook, Lino Becerra

**Affiliations:** 1 Center for Pain and the Brain, Harvard Medical School, Boston, Massachusetts, United States of America; 2 Department of Anesthesiology, Perioperative and Pain Medicine, Boston Children’s Hospital and Harvard Medical School, Boston, Massachusetts, United States of America; 3 Department of Radiology, Martinos Center for Biomedical Imaging, Massachusetts General Hospital and Harvard Medical School, Boston, Massachusetts, United States of America; 4 Department of Cardiology, Boston Children’s Hospital and Harvard Medical School, Boston, Massachusetts, United States of America; University of Medicine & Dentistry of NJ - New Jersey Medical School, UNITED STATES

## Abstract

The predictability of pain makes surgery an ideal model for the study of pain and the development of strategies for analgesia and reduction of perioperative pain. As functional near-infrared spectroscopy reproduces the known functional magnetic resonance imaging activations in response to a painful stimulus, we evaluated the feasibility of functional near-infrared spectroscopy to measure cortical responses to noxious stimulation during general anesthesia. A multichannel continuous wave near-infrared imager was used to measure somatosensory and frontal cortical activation in patients undergoing catheter ablation of arrhythmias under general anesthesia. Anesthetic technique was standardized and intraoperative NIRS signals recorded continuously with markers placed in the data set for the timing and duration of each cardiac ablation event. Frontal cortical signals only were suitable for analysis in five of eight patients studied (mean age 14 ± 1 years, weight 66.7 ± 17.6 kg, 2 males). Thirty ablative lesions were recorded for the five patients. Radiofrequency or cryoablation was temporally associated with a hemodynamic response function in the frontal cortex characterized by a significant decrease in oxyhemoglobin concentration (paired t-test, p<0.05) with the nadir occurring in the period 4 to 6 seconds after application of the ablative lesion. Cortical signals produced by catheter ablation of arrhythmias in patients under general anesthesia mirrored those seen with noxious stimulation in awake, healthy volunteers, during sedation for colonoscopy, and functional Magnetic Resonance Imaging activations in response to pain. This study demonstrates the feasibility and potential utility of functional near-infrared spectroscopy as an objective measure of cortical activation under general anesthesia.

## Introduction

Surgery results in nociceptor activation, inflammation at the surgical site, and nerve injury [1]. Nociceptor input can lead to central sensitization, defined as a reversible increase in the excitability and synaptic efficacy of neurons and circuits in central nociceptive pathways as well as reduced nociceptive inhibition [2, 3]. Central sensitization, by virtue of the abnormal perceptual response to normal sensory input, presents as hyperalgesia, allodynia, after sensations, and spread of pain to areas without discernible pathology. This topic has been reviewed elsewhere [1, 2, 3]. Consequences of central sensitization are plastic changes at a number of levels in the central nervous system, but especially the cortex, leading to altered and pathological behaviors referred to as maladaptive plasticity [4].

Individuals vary in their perception of pain and response to tissue injury, and different pain patterns may be seen in the perioperative period. It has been hypothesized that changes in pain sensitivity are an important contribution whereby severe, unrelieved acute postoperative pain may result in persistent postoperative pain [5, 3, 6]. As surgery is a common and predictable source of painful stimuli, it represents an ideal model for the study of pain and the development of strategies to guide analgesic administration and develop interventions to ameliorate persistent postoperative pain. However, objective and robust measures of nociceptive processing are a prerequisite to detect and prevent repeated nociceptive afferent discharges, central sensitization, and changes in brain systems [7].

Recent advances in brain imaging may be applied to the monitoring of perioperative nociception. Functional near-infrared spectroscopy (fNIRS) is a non-invasive, non-ionizing method for functional monitoring and imaging of brain hemodynamics [8]. It takes advantage of the low absorption of near-infrared light by biological tissues to measure changes in concentrations of oxygenated (HbO), de-oxygenated (HbR), and total hemoglobin (HbT) in the brain. Increased neuronal activity (activation) is associated with vascular dilation and increased cerebral blood flow such that the increase in cerebral O_2_ delivery is greater than the increase in cerebral O_2_ consumption. These hemodynamic fluctuations result in increased concentrations of HbO and HbT and decreased concentrations of HbR [9], creating the basis of functional magnetic resonance imaging (fMRI) and fNIRS. Functional NIRS has now been used for over 20 years to measure and map brain activation in humans for a broad range of applications [8], and the instrumentation and methodology has been reviewed [10]. Functional near-infrared spectroscopy has been shown to reproduce the same fMRI activations in response to pain [11, 12, 13]. Initial and more recent studies in healthy volunteers utilizing fNIRS found that noxious stimuli produced contralateral activation in the somatosensory cortex and a deactivation (negative change) in the frontal cortex, and that based on the temporal and spatial characteristics of the response in the frontal cortex it was possible to discern painful from innocuous stimulation [14,15]. The aim of this study was to evaluate the feasibility of fNIRS to measure the responses to noxious stimulation in the somatosensory and prefrontal cortices in patients under general anesthesia. We hypothesized that noxious stimuli applied under general anesthesia would produce the same patterns of hemodynamic activation in somatosensory and prefrontal cortices as that found in healthy awake volunteers.

## Materials and Methods

### Subjects

The Institutional Review Board at Boston Children’s Hospital approved the study and its protocol. Written informed consent was obtained from subjects over 18 and from parents if the participant was a minor. Signed consent forms where archived according to HIPPA/Hospital regulations and a copy was given to participants or her/his parents. Eight patients 12 years of age and older (mean (SD) 15.8 (4.3) years, four males) scheduled for catheter ablation of arrhythmias under general endotracheal anesthesia were enrolled. All subjects were right-handed. Exclusion criteria were structural heart disease, neurologic disease, diabetes, smoking, psychoactive medications, and scalp or hair not permitting adequate optical light detection.

### Imaging Equipment

**Fig 1:**
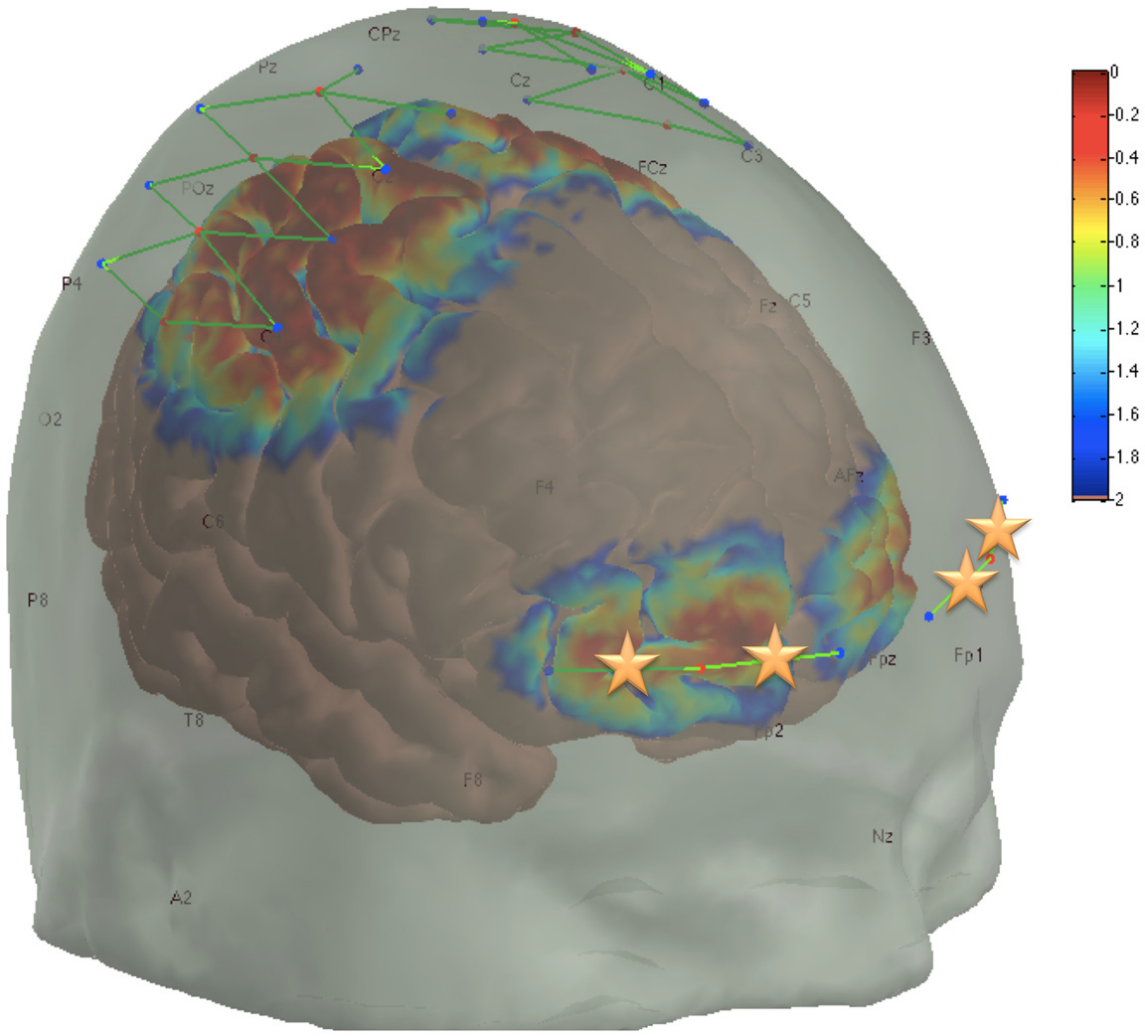
Sensor array and sensitivity profile. The fNIRS sensor array light sources (red dots), detectors (blue dots), and sensor channels (green lines) are shown at their intended locations. Although some shifting may occur for each subject, this has been shown to be sufficiently minimal in other studies where subject specific optode placements were measured. The sensitivity of the probe to detecting brain hemodynamics is shown as a logarithmic temperature plot ranging from 1.00 (0 dB, red) to 0.01 (-40 dB, blue) times the maximum sensitivity. The four frontal lobe channels used in this analysis are indicated with orange stars.

A multichannel continuous wave near-infrared imager (CW6, TechEn Inc., Milford, MA) was used to measure cortical activation. The system has previously been described in detail [16,17]. Briefly, two wavelengths of near-infrared light (690 nm and 830 nm) are emitted by 20 diode lasers and detected by 20 detectors (Fig 1). The sources and detectors are coupled to fiber optics, with each emitting fiber or detector constituting one optode. The optodes are 1-3mm in diameter, allowing them to be easily wiggled through the hair to make optical contact with the scalp. The source-detector separation is 3 cm, allowing for measurement of cortical activation at a depth of 1.5 cm [18]. The head probe holding the optodes covers the somatosensory and motor areas as well as the frontal cortex. Changes in cortical oxyhemoglobin (HbO) and dexoxyhemoblogin (HbR) are measured via differential absorption characteristics of the two wavelengths of near-infrared light [18].

### Protocol

The head probe was placed after midazolam premedication and prior to induction of general anesthesia. Signal quality was assessed by applying brush stimuli to the dorsum of the right hand for 5 seconds, with 15 seconds between, for 5 cycles. Anesthetic technique was standardized for all subjects. Anesthesia was induced with propofol and fentanyl and maintained with sevoflurane in air and oxygen. As a specific end-tidal sevoflurane concentration during ablation was not predetermined, sevoflurane was administered according to routine clinical practice. Neuromuscular blockade with vecuronium or rocuronium was maintained until the end of the procedure. No narcotics were administered beyond the immediate induction period. Intraoperative recordings were collected and markers placed in the data set, using an auxiliary data acquisition channel, for the timing and duration of each cardiac ablation event. The electrophysiologic study proceeded using the usual approach which consists of combinations of induced arrhythmia and pacing maneuvers to characterize and target the arrhythmia. These maneuvers frequently include a period of isoproterenol infusion. Radiofrequency or cryo applications are started for 5–10 seconds, and if deemed potentially successful continued for 60 seconds.

### Data Processing

Data analysis was performed using the open source software Homer2 [19]. Channels were pruned using the following exclusion criteria: insufficient signal strength (<80 dB), excessive signal strength (>140 dB), or insufficient signal-to-noise ratio (<2). Light intensity was then converted to optical density and sections of the data determined by the automatic motion artifact detection algorithm [tMotion: 0.5, tMask: 1.0, STDEVthresh: 50.0, AMPthresh: 5.00] were excluded using the time range [-5.0 20.0] (seconds). The data was then low-pass filtered below 0.5 Hertz and converted from optical density to chromophore concentration using a partial path factor of 6.0 for both frequencies. The data was then manually reviewed to identify periods of more than 120 seconds of artifact-free resting data in which time marks were semi-randomly placed as a control for comparison. Finally, the hemodynamic response function (HRF) was modeled using consecutive Gaussian temporal basis functions with a standard deviation of 1 second and their means separated by 1 second over the regression time range of -2 to 20 seconds, where time-zero is the initiation of the ablative process or a control time mark, as we have used previously [15]. The anatomical placement of each sensor channel is approximated in Fig 1. The prefrontal regions of interest from left to right are channels: 27, 28, 55, and 56, which correspond to left lateral, left central, right central, and right lateral locations, indicated with stars in Fig 1. Demographic and clinical parameters are presented as mean ± SD.

## Results

### Patients

Eight patients were studied, but due to poor signal quality in three patients, data from only five subjects was adequate for analysis. Mean age was 14 ± 1 years, weight 66.7 ± 17.6 kg, with a male: female ratio of 2:3. Anesthetic drugs administered intravenously comprised midazolam premedication 1.4 ± 0.3 mg, propofol 1.8 ± 0.4 mg/kg, fentanyl 1.5 ± 0.7 mcg/kg, and either vecuronium (n = 4) or rocuronium. No additional fentanyl was administered after the induction of anesthesia. End-tidal sevoflurane concentration was 2 ± 0.5% during application of the ablation lesions. As is the norm for catheter ablation of arrhythmias, there was significant variability in heart rate and to a lesser extent blood pressure during ablation. Isoproterenol was not administered during application of any ablation lesions. None of the recordings collected over the somatosensory cortex were of adequate quality for analysis.

### Lesions and NIRS Signal

**Fig 2:**
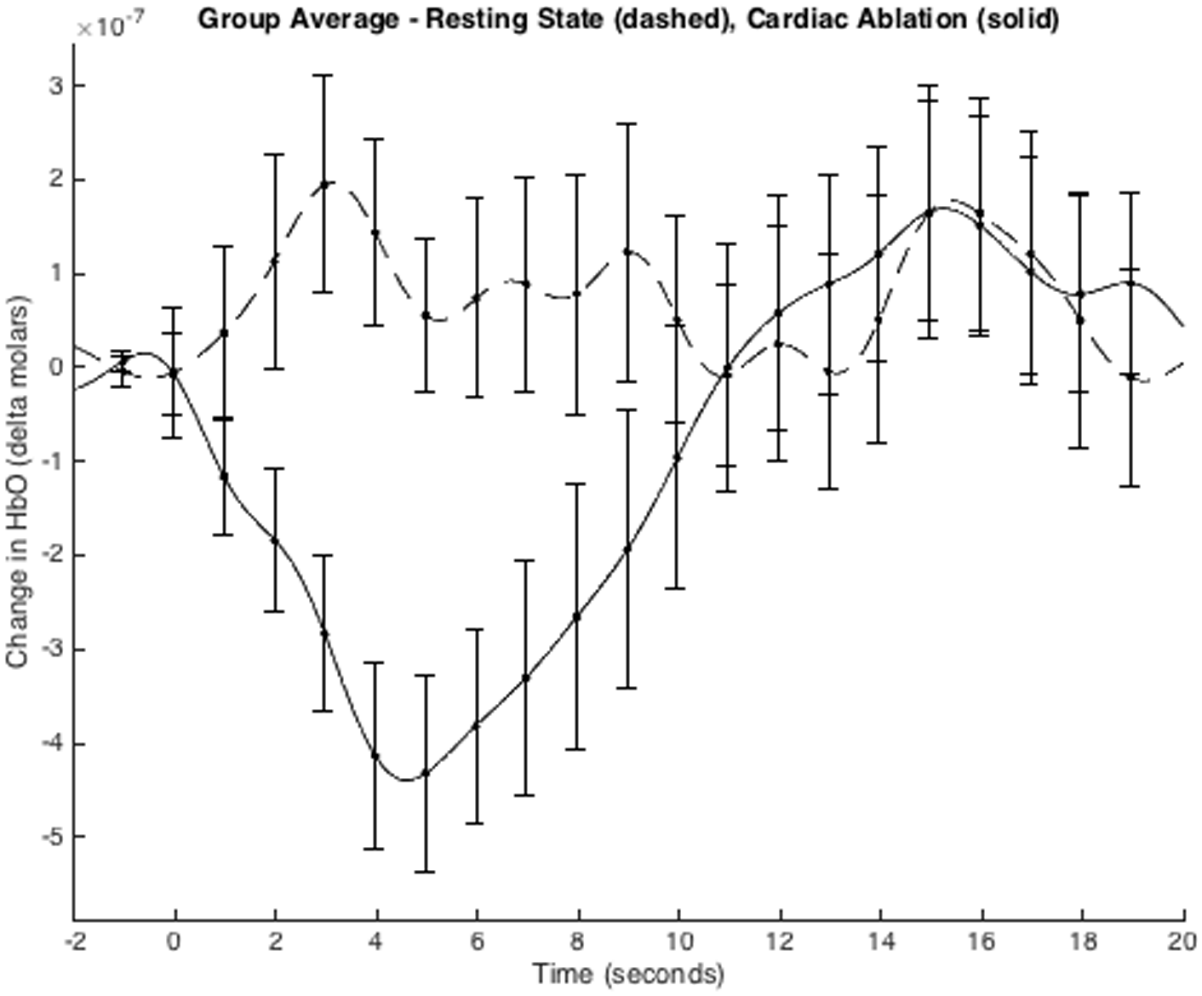
Group mean hemodynamic response function averaged across all frontal channels during cardiac ablation events. The average response for periods of rest (no ablations, less likely to be nociceptive) is represented with the dashed line and the response to cardiac ablations (more likely to be nociceptive) is represented by the solid line. Time-zero corresponds to either the initiation of the ablation processes or control time marks placed in artifact-free periods of rest, generally after induction of anesthesia but before any ablations have been performed.

Thirty ablative lesions were recorded for the five patients (Table 1). NIRS recordings acquired during radiofrequency ablation or cryoablation showed a HRF in the frontal cortex. Channels used for frontal cortical analysis overlie the superior frontal gyrus and medial frontal gyrus on each side. The HRF was characterized by a significant decrease in HbO concentration (paired t-test, p<0.05) with the nadir occurring in the period 4 to 6 seconds after application of the ablative lesion (Fig 2). Application of a single ablative lesion is in the order of 30 to 45 seconds.

**Table 1:** Patient demographics. The number of catheter-applied ablative lesions with processable signals that met the inclusion criteria for data processing and analysis are included in the right column.

Patient Number	Age (Years)	Weight (Kg)	Gender	Ethnicity	Diagnosis	Type of Ablation	Atrium	Number of Ablations
1	14	50.3	F	Caucasian	AVNRT	Radiofrequency	R	4
2	15	61	F	Caucasian	AVNRT	Radiofrequency + Cryo	R	4
3	12	61	M	Caucasian	WPW	Radiofrequency	R	4
4	16	101	M	Caucasian	AP	Radiofrequency	L	6
5	15	60	F	Hispanic	WPW + AVNRT	Radiofrequency	R	12

AVNRT, Atrioventricular Nodal Reentrant Tachycardia; WPW, Wolf-Parkinson-White syndrome; AP, accessory pathway; R, right atrium; L, left atrium

**Fig 3:**
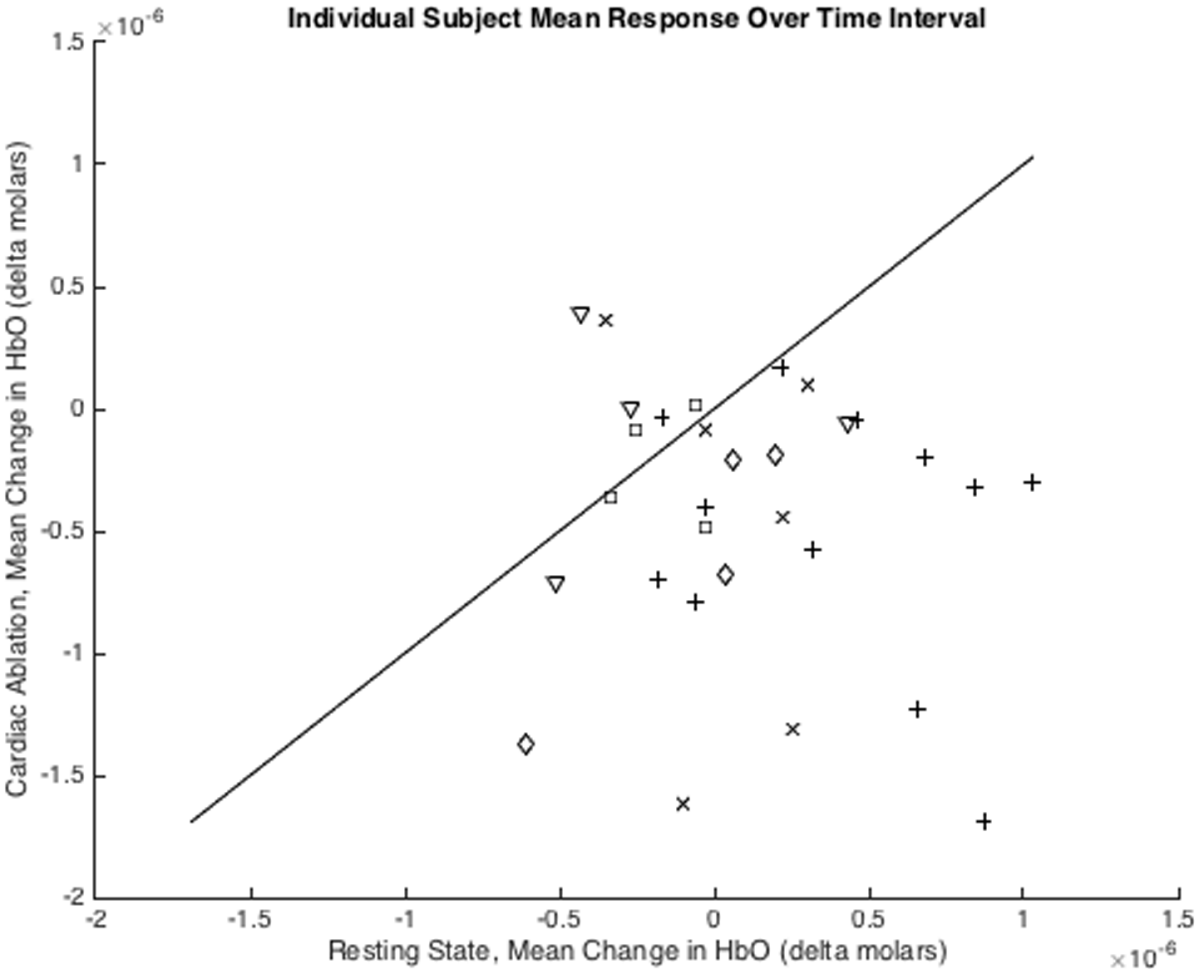
Individual Measures. The mean change in the oxygenated hemoglobin concentration (HbO) over the time period 4 to 6 seconds post ablation onset to differentiate the response to cardiac ablations (more likely to be nociceptive) versus periods of rest (less likely to be nociceptive). Plotting the mean response across the four frontal channels, for each of the 30 ablations that were recorded, shows that the decrease in HbO is greater than the paired resting period for the majority of ablation events (p = 0.0004). The difference between nociceptive and resting states is -5.34±7.38x10^-7 (mean ± standard deviation).

To investigate the frontal signals further, the HRF for each individual subject was averaged over the time interval 4 to 6 seconds post ablation onset (same method as used in Yücel et al., 2015 [15]), producing single metrics for the HbO response for each cardiac ablation event (n = 30). To evaluate the effectiveness of this method for differentiating between a cardiac ablation event (nociception) and the resting state (no or less nociception), paired t-tests were used to calculate the power for each channel (Table 2 and Fig 3). While channels 28, 55, and 56 all reached significance, channel 27 did not. This is likely due to the decreased number of ablation events measured on this channel as a result of channel 27 not passing the data quality assurance procedures performed by Homer2 for patient #5 (12 of the ablation stimuli).

**Table 2:** Results of paired t-tests for each channel. Significance was reached for three of the channels individually for within-event identification of whether the change in HbO concentration is the result of a processable ablation or a stimulus-free state.

	Channel 27 (left, lateral)	Channel 28 (left, central)	Channel 55 (right, central)	Channel 56 (right, lateral)
t-test Result	p = 0.2306	p = 0.0010	p = 0.0015	p < 0.0001
Number of Ablations	n = 18	n = 30	n = 30	n = 30
Mean Delta (moles)	-2.15x10^-7	-5.20x10^-7	-5.08x10^-7	-6.21x10^-7
Standard Deviation of Delta	7.41x10^-7	7.75x10^-7	7.95x10^-7	6.59x10^-7

## Discussion

In this study, we demonstrate the feasibility of using fNIRS to measure cortical responses to noxious stimulation in patients under general anesthesia. The cortical signals produced by catheter ablation of arrhythmias mirror those seen with noxious stimulation in healthy volunteers and fMRI activations in response to pain. Our results support the notion that NIRS has a potential utility as an objective measure of nociception and pain. Specifically, we report that the frontal cortical signals detected by NIRS are temporally related to the cardiac ablative lesions, suggesting that catheter ablation of arrhythmias produces changes in brain activity associated with pain. Importantly, the NIRS signals we found are similar to those produced by painful stimuli in awake, healthy volunteers [14, 20,21, 15], during sedation for colonoscopy Becerra et al PMC4794375, and fMRI activations in response to pain [11, 12, 13,22].

Patients undergoing cardiac ablation have described the procedure as painful; indeed the majority of patients complain of chest pain during, [23–25] and occasionally several days following, the operation. Thus, there is clinical evidence the ablation produces pain. Ablation by radiofrequency destroys tissue, which activates nociceptive fibers in a similar manner to a burn [24,26]. Therefore, the source of nociceptive afferent drive has a clinical and neurophysiological basis in patients undergoing catheter ablations for control of their cardiac arrhythmias. Pain pathways from the heart are thought to originate in sympathetic fibers that synapse on the spinothalamic tract neurons in the upper thoracic and lower cervical segments and on second order neurons that project to the thalamus. Additional fibers from the area of injury enter the brain via the nucleus of the solitary tract and connect with higher order regions such as the amygdala [27]. From these locations, third order neurons project to cortical regions, such as the prefrontal cortex [28]. The frontal lobe also receives input from a number of brain regions involved in nociceptive processing [29,30] and sends efferent projections to areas such as the periaqueductal gray to modulate (inhibit under healthy conditions) nociceptive activation of cells in the dorsal horn of the spinal cord. Conceptually, this provides a model for the activation profile we observe in the frontal regions, which is based on a balance between increased activation (resulting from afferent nociceptive input) and decreased activation (resulting from inhibitory processes in these regions).

### The Issue: Surgical Intervention and Measures of Pain

Catheter ablation using radiofrequency energy is associated with pain, even with the administration of sedative and analgesic agents [31, 26, 32]. The mechanisms and pathways of cardiac pain are complex and have recently been reviewed [27]. Briefly, nociceptive information is transmitted by (1) cardiac visceral afferent fibers travelling with the sympathetic nerves via the dorsal root ganglia into the spinal grey matter and ascending via the contralateral spinothalamic tract (STT) to the thalamus; and (2) vagal afferents via the nodose ganglia to the nucleus tractus solitarius in the brainstem. Above the level of the thalamus, sensory information is relayed not only to the somatosensory cortex but also to the cingulate gyrus, insula, amygdyla, hypothalamus, and prefrontal cortex. Activations of the prefrontal cortex mediate part of the cognitive and emotional dimensions of pain processing [33, 34].

Autonomic responses (changes in heart rate, blood pressure, and pupillary size) via the nociceptive medullary autonomic circuit, rather than direct measures of consciousness and analgesia, are used as surrogates to guide drug administration during general anesthesia [35]. Hence, intermittent or continuous barrages of nociceptor-induced neural activity during (and after) surgery can result in peripheral and central pain sensitization with pain hypersensitivity, changes in brain activity, and persistent postsurgical pain [7, 2,5]. Pain has been reported as a component of intraoperative awareness, but the frequency and relationship to significant psychological sequelae is inconsistent [36, 37, 38].

Assessing pain in individuals not able to communicate (e.g. neonates, infants, general anesthesia, coma, or following stroke) might be very difficult due to the lack of non-verbal objective measures of pain. The consequences of ongoing pain include peripheral and central sensitization with amplification of the pain and allodynia. Additionally, there are changes in brain systems (‘centralization of pain’) affecting emotional and cognitive processing [7]. Near-infrared spectroscopy (NIRS) being a portable, non-invasive and inexpensive method of monitoring cerebral hemodynamic activity has the potential to provide such a measure. In a prior study we have used functional NIRS to evaluate brain activation to an innocuous and a noxious electrical stimulus on healthy human subjects. The painful and non-painful stimuli can be differentiated based on their signal size and profile [15]. Furthermore, the signal was distinguishable from a skin sympathetic response to pain that tended to mask it. Our results support the notion that functional NIRS has a potential utility as an objective measure of pain processing.

### NIRS Measures in Response to Ablation of Arrhythmias

Although the pattern of change in HbO concentration in response to cardiac ablation resembles that found in awake, healthy volunteers [15], the time to peak change was shorter (by about 50%) in our patients. Possible explanations for this include the effects of anesthesia on cerebral hemodynamics, the location of the stimulus (heart versus hand), the type of stimulus, or a combination of these effects. Another important aspect of the observed signal is the amplitude. The peak values for the HbO response to cardiac ablation range from approximately -1.7 to 0.5 micro Moles, with a mean value around -0.44 micro Moles. This is substantially larger than the response observed in our healthy subject studies, which utilize short separation regression to remove systemic effects such as the skin response. However, based on prior observations, we have verified that the frontal cortex response, without the use of a regression measure, also resembles the results reported in this study with a similar increase in amplitude [15].

The somatosensory cortex responses during cardiac ablation are also of great interest. Due to procedural limitations in this study, NIRS channels over the somatosensory cortex often deviated from their preoperative configuration, reducing the signal to noise ratio below acceptable levels. We have reason to be concerned that systemic activity could be producing the hemodynamic response function in the prefrontal cortex. Short separation channels were utilized during the collection of this data set, but were only located at medial somatosensory locations, which are not useful for regression of signals in the prefrontal cortex. To overcome this limitation and enhance our confidence in the data, a second cohort of cardiac ablation patients is being studied with a different sensor positioning system utilizing short separation channels located adjacent to all channels. This will allow us to eliminate any systemic effects that may be present in the scalp or skull over the prefrontal cortex and rule out the possibility of a sympathetic response. We also expect to be able to detect a differentiable response in the somatosensory cortex region [15], which will corroborate our results in the prefrontal cortex. There is some uncertainty over where heart pain is processed in the brain, with the possibility that the insular lobe is responsible for both heart control and sensation, but there is support that a more accessible cortex location may be available [39].

### The Frontal Lobe and Pain

Our study focused on measures of pain in the frontal lobe, an area that is frequently reported in experimental and pain studies. The specific regions are the superior medial and mid-frontal lobe.

The frontal lobe has been implicated in a number of functional processes including: (1) defining future processes that may result from current actions and defining relative choice outcomes (viz., good/bad; aversive/rewarding) between these actions; (2) defining responses in a social context; (3) defining relative congruency of processes (viz., objects or proceedings); and (4) long-term emotional memories. In this study we evaluated changes in the medial frontal lobe [40, 41, 42, 43] that has attributed functions including those in fear and extinction [44]. How are these functions altered by nociceptive signals during unconsciousness associated with anesthesia? First, there is accumulating evidence that there may be non-conscious or unconscious processing of fear by the medial prefrontal cortex [45, 46]. While disturbances of network properties (including those in the medial prefrontal cortex) during impaired consciousness (viz., coma) or anesthesia [47], prior work in animals under general anesthesia show that nociceptive signals activate somatosensory pathways [48, 49, 50]. Second, the connectivity of the medial prefrontal cortex has been studied in rats, monkeys and humans and data supports a major cortical output from the region to visceromotor structures in the hypothalamus and brainstem, the basal ganglia (accumbens, putamen and ventromedial caudate) and mediodorsal thalamus [51, 52]. Its role in pain is supported by a number of clinical and preclinical studies: (1) the regions noted above are all regions activated by nociceptive signals in human fMRI studies and notably the region is predictive for hyperalgesia and anti-hyperalgesia [53]; the latter is of note since we suggest that the region may be a marker for analgesia; (2) alterations in channel function are reported in in the region in neuropathic pain produced by spared nerve injury in rats [54]; and pain produces deactivation of the medial prefrontal cortex (mPFC) which is part of or closely associated with the s-mPFC that alters glutamate and GABA receptors [55] in a manner that may contribute to pain induced deactivation; (3) Consistent with an inhibitory effect, medial prefrontal areas project to the periaqueductal gray [56], a region involved in descending modulation and perhaps involved in adaptation or habituation of a repetitive fNIRS stimulus we have previously reported [15]. Under normal conditions, GABAergic inhibition may thus be at play, resulting in decreased evoked activity in mPFC neurons—and perhaps consistent with the decreased fNIRS signal observed in our study.

We propose that during incomplete analgesia, nociceptive signals activate pathways that include spinothalamic/spinoparabrachial-amygdala pathways and that these areas (thalamus and amygdala) relay the information to the medial prefrontal cortex. The decreased fNIRS signal is putatively related to enhanced GABAergic processing (see [55]). Its unconscious processing continues presumably because of the relative depth of anesthesia. Thus represents evaluation of ‘*current action*’ (including the aversive emotional nature of nociception) and while interrogating this information attempting to define a course of action, consistent with known function of the superior medial prefrontal cortex (s-mPFC).

### Measures of Pain and Anesthesia

Although definitions and goals of the anesthetic state vary among anesthesiologists, important components of general anesthesia include lack of experience of surgery (unconsciousness or disconnected consciousness, amnesia), immobility, and nociceptive blockade [57]. The relationship of memory to level of consciousness is complex, and both inhalation and intravenous anesthetics can cause memory blockade at doses considerably lower than those required for loss of consciousness and immobility [58]. Monitoring brain states under general anesthesia and sedation is presently directed at assessment of the level of consciousness and prevention of awareness. This is still performed by interpretation of physiologic parameters and to a lesser extent by EEG-derived indices and changes in the unprocessed electroencephalogram (EEG).

Although attempts to develop an analgesic state monitor in response to sympathetic stimulation [59] or evoked potentials [60, 47] are ongoing, a technique which can detect and reduce cerebral nociceptive input would be superior. Validation of this technique to measure pain processing would allow testing of the hypothesis that reduction in nociceptive processing during anesthesia would allow for development of strategies to improve analgesia in the perioperative period [2,5,7].

### Caveats

Hemodynamic effects initiated by neuronal activity are considerably smaller in magnitude than the background variations in blood oxygenation and volume caused by systemic activities (e.g. cardiac rhythm, respiration, and blood pressure oscillation). To improve the ability to identify single events, a regression technique such as using short separation optodes [61], in addition to our sensor array, should be used. Additionally, near-infrared spectroscopy is dependent on consistent contact with the medium being imaged. Excessive patient movement can cause both loss of contact with the scalp as well as movement within the cerebral fluid. Loss of contact results in sudden changes in received light intensity. Signal processing methods are now being developed to correct for these types of artifacts [62].

These results do not take into account any changes that occur due to the influence of pharmacologic agents on hemodynamic response to stimuli or brain function in general [63, 64, 65, 66]. Cortical activity monitored in healthy, awake individuals through NIRS may not provide a direct comparison to individuals undergoing cardiac ablation, because a pain signal observed in patients under anesthesia may exhibit variations in amplitude or time of onset of activity, as well as other factors not present in the control data.

## Conclusions

The findings of this study indicate that detection of cortical nociceptive activation signatures is possible with fNIRS in patients under general anesthesia. Specifically, this suggests that further research could provide a measure of pain through fNIRS measures in the prefrontal cortex. If successful, this would create a tool that a variety of pain related fields could use for objectification of pain as well as pain detection in populations with no means of communicating pain intensity. This would almost certainly improve the study of pain as well as our ability to provide perioperative pain relief.
